# Mycobacterial *tlyA* gene product is localized to the cell-wall without signal sequence

**DOI:** 10.3389/fcimb.2015.00060

**Published:** 2015-08-21

**Authors:** Santosh Kumar, Ekansh Mittal, Sapna Deore, Anil Kumar, Aejazur Rahman, Musti V. Krishnasastry

**Affiliations:** Membrane Biology, National Centre for Cell Science, Savitribai Phule Pune UniversityPune, India

**Keywords:** TlyA gene product, signal sequences, cell-wall localization, membrane fractions, outer membrane vesicles

## Abstract

The mycobacterial *tlyA* gene product, Rv1694 (MtbTlyA), has been annotated as “hemolysin” which was re-annotated as 2′-O rRNA methyl transferase. In order to function as a hemolysin, it must reach the extracellular milieu with the help of signal sequence(s) and/or transmembrane segment(s). However, the MtbTlyA neither has classical signals sequences that signify general/Sec/Tat pathways nor transmembrane segments. Interestingly, the *tlyA* gene appears to be restricted to pathogenic strains such as H37Rv, *M. marinum, M. leprae*, than *M. smegmatis, M. vaccae, M. kansasii* etc., which highlights the need for a detailed investigation to understand its functions. In this study, we have provided several evidences which highlight the presence of TlyA on the surface of *M. marinum* (native host) and upon expression in *M. smegmatis* (surrogate host) and *E. coli* (heterologous host). The TlyA was visualized at the bacterial-surface by confocal microscopy and accessible to Proteinase K. In addition, sub-cellular fractionation has revealed the presence of TlyA in the membrane fractions and this sequestration is not dependent on TatA, TatC or SecA2 pathways. As a consequence of expression, the recombinant bacteria exhibit distinct hemolysis. Interestingly, the MtbTlyA was also detected in both membrane vesicles secreted by *M. smegmatis* and outer membrane vesicles secreted by *E. coli*. Our experimental evidences unambiguously confirm that the mycobacterial TlyA can reach the extra cellular milieu without any signal sequence. Hence, the localization of TlyA class of proteins at the bacterial surface may highlight the existence of non-classical bacterial secretion mechanisms.

## Introduction

The causative agent of human tuberculosis disease, *Mycobacterium tuberculosis*, uses novel mechanisms for evasion of host responses for its intracellular survival (Berry et al., 2013; Cambier et al., 2014). Often bacterial pathogenicity depends upon the ability to stash virulence factors which can be either displayed at the bacterial cell surface or secreted into extracellular milieu in addition to direct injection into the target cell (Tseng et al., 2009). In bacteria, protein export across the cytoplasmic membrane represents the first step in the delivery of proteins to the cell envelope or extracellular space. Among many transport mechanisms, two conserved systems account for a majority of the protein export: the general secretion (Sec) and the Twin-arginine translocation (Tat) pathways (Lee et al., 2006; Palmer and Berks, 2012; Beckwith, 2013; Denks et al., 2014). The Tat and Sec systems recognize amino terminal signal sequences for their transport across the cytoplasmic membrane and also the proteins being exported may remain associated with the cell envelope or may get secreted into the extra-cellular milieu depending upon the function of the protein. Although, the signal sequences of Sec and Tat substrates share a seemingly similar domain structure, the Tat substrates can be distinguished with the help of twin arginine motif, “R-R-X,” where X can be any amino acid. The two pathways, *viz*. Sec and Tat also differ in their mode of transport. Sec substrates are translocated across the cytoplasmic membrane in an unfolded state, whereas Tat substrates are translocated in a folded conformation (Berks, 1996; DeLisa et al., 2003; McDonough et al., 2008). In mycobacteria, apart from the Sec and Tat systems, it has another secretion system, known as Esx-1, which secretes Esat6 and Cfp10. The Esx-1 module is also present in the fish pathogen *Mycobacterium marinum* and non-pathogenic species *Mycobacterium smegmatis* (Abdallah et al., 2007; Champion and Cox, 2007; Simeone et al., 2015). While the Esx-1 has been shown to be responsible for virulence in *M. tuberculosis* and *M. marinum*, the same is responsible for conjugation in *M. smegmatis* (Gao et al., 2004; Converse and Cox, 2005). Hence, a comprehensive understanding of protein secretion pathways is essential not only for delineating the mechanism of translocation but also the substrates that utilize these modules for exiting into extra cellular milieu. In this regard, an earlier observation has suggested that expression of *tlyA* gene of *M. tuberculosis* (Rv1694; MtbTlyA) in *M. smegmatis* has resulted in a significant increase in contact dependent hemolysis of red blood cells (King et al., 1993; Wren et al., 1998). However, the basis for the increase in hemolysis has not been understood or defined because the *tlyA* gene product of *M. tuberculosis* was initially said to be a hemolysin but it has been later re-annotated as an S-Adenosylmethionine dependent, rRNA methylase, whose function is to methylate the nucleotides C1409 and C1920 of 16S and 23S rRNA respectively (Johansen et al., 2006). Methylation of rRNA reduces the translational ability in the presence of Capreomycin, a second generation antibiotic. In contrast to this, the purified MtbTlyA has been shown to possess hemolytic activity through formation of stable oligomers on RBC of both rabbit and human as well as on phagosomes of mouse macrophages (Rahman et al., 2010). These two properties are not only diverse but starkly contrasting i.e., as a hemolysin the TlyA must be a cell-wall attached entity or as an rRNA methylase, it must be an intracellular protein. Hence, there is a need for a detailed investigation of the pathway the TlyA like proteins utilize to reach the extra-cellular milieu. In this study, we have used *M. marinum* (native host), *M. smegmatis* (as a surrogate host) *and E. coli* (as a heterologous host) to study the transport of TlyA across the cell membrane. Our observations reveal that in both native and recombinant hosts, the TlyA can reach the bacterial surface in functional form and such a sequestration, in principle, may aid the intra-cellular survival mechanisms.

## Materials and methods

The antibodies to HBHA (NR-13804), GroEL (CS-44), DnaK (IT-40) were obtained from BEI resources, USA. TlyA immunization protocols in mice and rabbit were approved by the Institutional Animal Care Committee of National Centre for Cell Science, Pune.

### Cloning and expression of TlyA

MtbTlyA (Rv1694) was cloned in two vectors *viz*. pT7Nc and pET28a+ between the sites NcoI and HindIII with and without a C-terminal 6-histidine tag (Rahman et al., 2010). We have also cloned the MtbTlyA in pMyNT vector for expression in *M. smegmatis* (Noens et al., 2011). All the constructs used in this study have been verified by di-deoxy nucleotide sequencing.

### Titration of inducer

The expression of MtbTlyA in *M. smegmatis* was judged by varying the induction strength i.e., Acetamide from 0.2 to 0.001%. We have set the inducer concentration at 0.001% for all experiments described here to ensure sub-optimal expression of the TlyA.

### Expression and purification of TlyA

The *E. coli* expressed MtbTlyA carries a carboxy terminal 6-histidie tag which was used for purification with the help of Ni-NTA resin (Qiagen, Germany), essentially following the procedure reported by us earlier (Rahman et al., 2010). The purity of the protein was routinely assessed by 12% SDS-PAGE. The purification attempts usually yield about ~0.7–1 mg of TlyA from a 2 liter culture volume. The TlyA purified from both *M. smegmatis* and *E. coli* was verified by MS-MS sequencing.

### Hemolysis assay of purified MtbTlyA

Purified MtbTlyA was diluted in Sodium phosphate buffer (25 mM, pH 7.4) and NaCl (150 mM) buffer, mixed with 1.5% rabbit red blood cells (rRBC) and incubated at room temperature (25°C) for 24 h. After centrifugation, the absorbance of the supernatant at 540 nm was measured. Water was used as a control for total RBC lysis. The per cent hemolysis was estimated as follows: Hemolysis (%) = [(A_540_ of sample – A_540_ controls) × 100]/(A_540_ of Water lysed sample).

### Bioinformatics analysis

The grand mean of hydropathy (GRAVY) score was calculated using the PROTPARAM tool (http://us.expasy.org/tools/protparam.html). Transmembrane regions were predicted using TMHMM (http://www.cbs.dtu.dk/services/TMHMM) and TMPRED (http://www.ch.embnet.org/software/TMPRED_form.html). Cellular localization and signal sequences were predicted from CBS Prediction Servers (http://www.cbs.dtu.dk/services/signal_p). Tat signal sequence was predicted using CBS Prediction Servers Tat P (http://www.cbs.dtu.dk/services/TatP) and TatFind (http://signalfind.org/tatfind.html).

### RNA isolation, cDNA synthesis and RT-PCR

Non-transformed *M. smegmatis* and TlyA transformed bacteria were grown in 7H9 Middlebrook medium till mid-log phase and RNA was extracted as described (Kurthkoti and Varshney, 2010). The total RNA preparation was treated with turbo DNase at 37°C for 15 min. The absence of DNA was confirmed by PCR using the DNase treated RNA as template. The RNA (~3 μg) was used for cDNA synthesis by using Superscript II RT system (Invitrogen). The reaction mixture containing RNA (3 μg) and of Oligo-dT primer (250 nmol) was heated to 65°C for 5 min and quick chilled on ice for 5 min. The primer was annealed to the template at 60°C for 5 min, chilled, and mixed with a cocktail containing, dNTPs (10 mM), MgCl_2_ (25 mM), RNasin and Superscript II RT (50U) to a total volume of 10 μl. The primers annealed to RNA template were extended at 40°C for 1 h and heated at 70°C for 10 min to terminate the reaction. Aliquots of cDNAs (25 ng) were used as templates for RT-PCR with the forward and reverse primers (50 pmol each) specific to MsTlyA and MtbTlyA shown in Table 1.

**Table 1:** **List of Bacterial Strains, plasmid and Primers used in the present study**.

**Bacterial strains**	**Description/Purpose**	**Source/References**
***E. coli***		
DH5α	Cloning	Invitrogen, USA.
BL21 (DE3)	For expression of TlyA	Invitrogen, USA.
***M. smegmatis***		
mc^2^155	Wild type	Noens et al., 2011
MB692	mc^2^155, Δ*TatA*	McDonough et al., 2005
JM567	mc^2^155, Δ*TatC*	McDonough et al., 2005
NR116	mc^2^155, Δ*SecA2*	Gibbons et al., 2007
*M. marinum*	Wild type	Gao et al., 2004
**PLASMIDS**		
pET28a+	For expression in BL21 (DE3)	Novagen, USA.
pMyNT	For expression in *M. smegmatis* mc^2^155	Noens et al., 2011
pET28a+	For expression of GFP	Mittal et al., 2014
**PRIMERS**		
** MtbTlyA**		
Forward		5′-TATATATCCATGGCTCGACGTGCCCGCG-3′
Reverse		5′-TATATAGAATTCTTACGGGCCCTCGCTAATCGC-3′
** MsTlyA**		
Forward		5′-TGAGGAGCTAGCGTGGCACGGCGAGC 3′
Reverse		5′- TCATAAGCTTTTGCGGCCCTTCC 3′

### Bacterial strains and culture conditions

All the bacterial strains, vectors used in this study are described in Table 1. *M. marinum, M. smegmatis* were maintained in 7H9 Middlebrook medium as described earlier (Noens et al., 2011). For protein expression, *M. smegmatis* mc^2^155 was grown in 7H9 Middlebrook medium supplemented with glucose (0.2% w/v), Tween-80 (0.05% v/v), and Hygromycin B (50 μg/ml) in a shaker incubator maintained at 37°C till OD_600_ ~0.5. The culture was then induced with Acetamide (0.001% w/v) and harvested at 7000 × g. *E. coli* expression was achieved in LB medium as reported earlier (Rahman et al., 2010).

### Contact dependent hemolytic assay

Contact dependent hemolytic assay was performed as described previously (Rahman et al., 2010). Briefly the bacterial pellet, obtained as described above, was re-suspended in Sodium Phosphate buffer (25 mM, pH 7.4) and NaCl (150 mM) and washed twice with the same buffer. Mock transformed and TlyA expressing bacteria (2 × 10^7^) were mixed with RBC (1%) briefly centrifuged to establish physical contact with the RBC and incubated for 24 h at room temperature. At the end of incubation, the samples were centrifuged and the absorbance of the supernatant was measured at 540 nm. Water was used to obtain total RBC lysis value.

### LC-MALDI-MS/MS analysis

**Figure 1:**
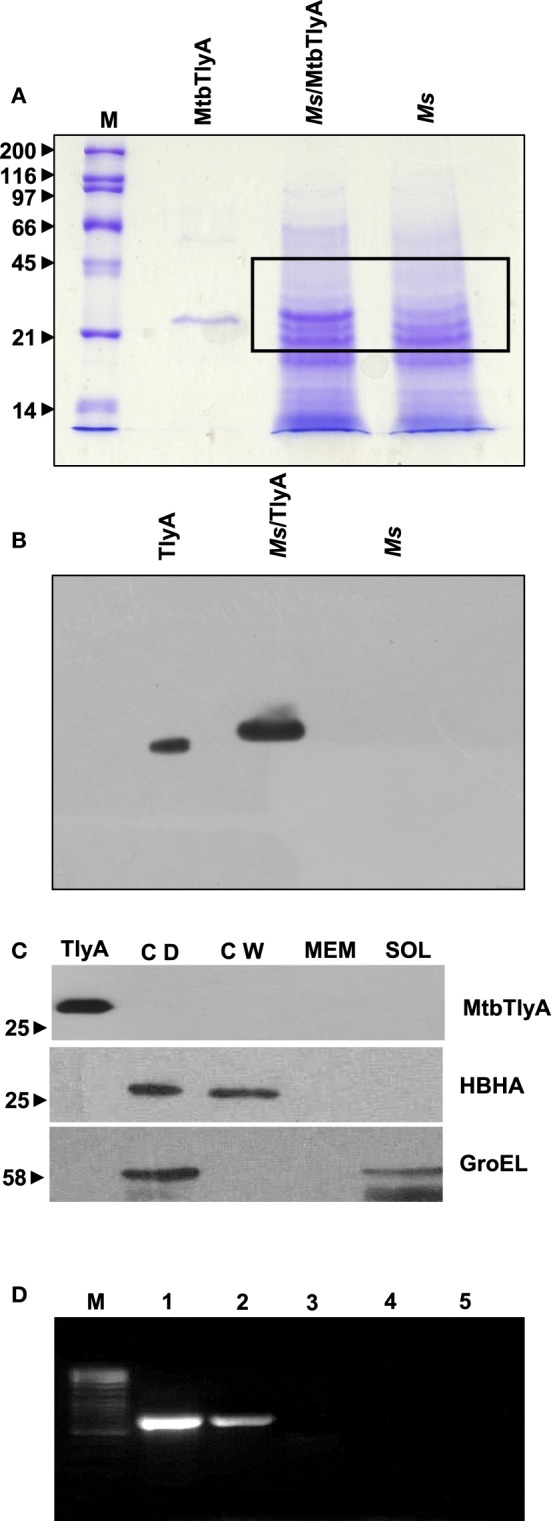

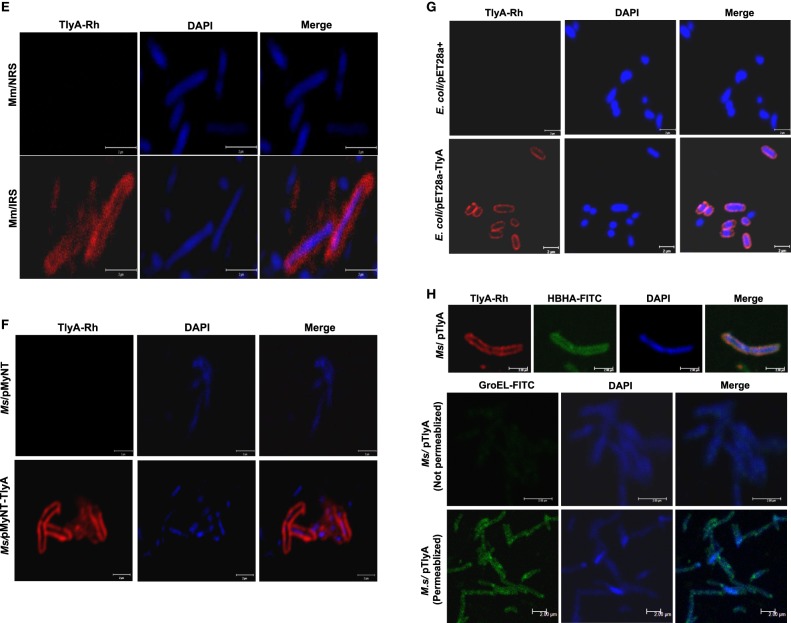
**(A) Expression of TlyA in ***M. smegmatis***:** *M. smegmatis*/TlyA and *M. smegmatis* (2 × 10^7^) were processed for SDS-PAGE (12%) and stained with Coomassie brilliant blue G-250. The lanes indicated with M, MtbTlyA, Ms/MtbTlyA, and Ms respectively represent Molecular weight markers (kD), Purified MtbTlyA protein from *E. coli*, whole cell lysate of *M. smegmatis* expressing MtbTlyA, wild-type *M. smegmatis*. The MtbTlyA isolated from *E. coli* expression is 10 amino acids shorter than the *M. smegmatis* expressed protein. Boxed region (MW range ~21–45 kD) was subjected to LC-MS analysis (Supplementary Tables 1–4). **(B) Immunoblot of TlyA expressing ***M. smegmatis***:** *M. smegmatis* (2 × 10^7^) or pMyNT-MtbTlyA were processed for SDS-PAGE and probed with anti-TlyA antibody. The lane indications are as follows: TlyA: Purified MtbTlyA from *E. coli*. Ms/TlyA: Whole cell lysate of *M. smegmatis* expressing MtbTlyA; Ms: wild-type *M. smegmatis*. **(C) MsTlyA is undetectable in ***M. smegmatis***:** *M. smegmatis* wild type was fractionated as described in methods section and examined for the presence of MsTlyA (MSMEG_3751) by using the same antibody used in **(B)**. The lane markings represent the samples as follows: TlyA, Purified MtbTlyA from *E. coli*; CD, cells debris obtained after 3000 g value; CW, cell-wall fraction obtained after 27000 g; MEM, pellet of the membrane fraction obtained after 100,000 g centrifugation; SOL, supernatant of the 100,000 g centrifugation. The top panel was developed with anti-TlyA antibody, middle panel represents the identification of MsHBHA obtained with anti-HBHA antibody (BEI resources), and the bottom panel represents the MsGroEL obtained with anti-GroEL antibody (BEI resources). **(D) Detection of ***tlyA*** transcript:** RT-PCR of mRNA from MtbTlyA transformed and non-transformed *M. smegmatis* was done to detect the *tlyA* transcript as described in methods section. The lanes indicate: M: DNA ladder; Lanes 1–5 respectively represent the RT-PCR products using the DNA templates of pMyNT/*tlyA* vector (positive control), cDNA of *M. smegmatis*/*tlyA*, cDNA of non-transformed *M. smegmatis*, RNA of transformed *M. smegmatis*/*tlyA* and RNA of non-transformed *M. smegmatis*. **(E) Visualization of TlyA on ***M. marinum***:** Thin smears of *M. marinum* were visualized in confocal microscope after staining with rabbit anti-TlyA antibody and Rhodamine-anti-rabbit-IgG. The top and bottom panels represent the staining of *M. marinum* with normal rabbit serum (NRS) and immune rabbit serum specific to MtbTlyA (IRS) respectively. The panels, left, middle, and right represent the signal positive to MmTlyA, DAPI for bacterial staining and merged panels of MmTlyA and DAPI. Panels shown are a representative of one the several independent visualizations. **(F,G) Visualization of TlyA in ***M. smegmatis*** (F) and ***E. coli*** (G)**: The bacteria and the constructs are indicated on the left side of the panels. The primary antibody used is rabbit anti-TlyA antibody and secondary antibody was Rhodamine-anti-rabbit-IgG. The middle panels represent the DAPI staining. The merged panels are overlap of Rhodamine and DAPI channels The white bar represents 2 μm. Panels shown are a representative of one the several independent visualizations. **(H) Dual stating of TlyA transformed ***M. smegmatis***:** TlyA was visualized with anti-TlyA antibody and Rhodamine anti-rabbit antibody (TlyA-Rh). HBHA was visualized with mouse monoclonal anti-HBHA antibody and with FITC-anti-mouse-IgG (HBHA-FITC). DAPI labeled panels was obtained by staining of the bacteria with DAPI. The merged panels are overlap of TlyA-Rh, HBHA-FITC, and DAPI channels The white bar represents 2 μm. Panels shown are a representative of one the several independent visualizations. Intracellular staining of TlyA transformed *M. smegmatis*: TlyA transformed *M. smegmatis*, without (middle) and with (bottom) permeabilization, were stained for GroEL with mouse monoclonal anti-GroEL antibody and anti-mouse-IgG-FITC. DAPI labeled panel indicates the staining of the bacteria DAPI. The merged panels are overlap of FITC and DAPI channels The white bar represents 2 μm. Panels shown are a representative of one the three independent visualizations.

LC-MS analysis of the boxed region in Figure 1A was performed as per the mass spectrometry facility of NCCS using protocols published earlier (Målen et al., 2007; Prados-Rosales et al., 2011; Reddy et al., 2015).

### Microscopic visualization of TlyA

*M. marinum*, TlyA expressing *M. smegmatis or E. coli* were pelleted, washed several times with PBS and re-suspended in the same buffer followed by incubation with (1:100) immune rabbit serum raised against TlyA or other primary antibodies for 1–2 h on ice and washed thrice with PBS. After washing the bacteria were further incubated with appropriate fluorophore tagged secondary antibodies (1:100 dilution) for 1 hr and with DAPI for 10 min. A thin smear was made on the glass slides for confocal microscopy. Intracellular staining of TlyA transformed *M. smegmatis* was achieved by incubating the bacteria in Triton X-100 (0.1%) and Lysozyme (2 mg/ml) prior to primary antibody incubation keeping the rest of the procedure identical.

### Limited proteolytic digestion of TlyA expressing bacteria

Proteinase K accessibility of the TlyA was examined as described previously (Delogu et al., 2004). TlyA expressing bacteria were grown as described above and the bacterial pellet was re-suspended in PBS. Four aliquots of 200 μl (2 × 10^8^) were taken and three aliquots were treated with Proteinase-K (Promega, USA) to a final concentration of 100 μg/ml and all four tubes were incubated at 37°C. At indicated times aliquots were centrifuged at 7000 × g for 10 min at 4°C. Pellet and supernatants were electrophoresed on 12% SDS–PAGE and immune-probed with anti TlyA antibody and hemolytic assay was performed as described (Rahman et al., 2010).

### Subcellular fractionation of TlyA

Separation of cell-wall, soluble and membrane fractions of *M. marinum, M. smegmatis*, and *M. smegmatis/*TlyA was achieved by following the procedures published earlier (Gibbons et al., 2007; McDonough et al., 2008). Briefly, bacterial pellets (1 gm wet weight) were re-suspended in Tris buffer (50 mM, pH 8.0), NaCl (150 mM), and pulse sonicated for 20 min (30 s interval for every 30 s duty cycle) using Sonics Vibra m cell. Cell debris and unbroken cells were removed by centrifugation at 3000 × g for 20 min at 4°C. The resultant supernatant was further centrifuged at 27000 × g for 30 min at 4°C. The pellet is referred as the cell-wall fraction and the supernatant was subjected to ultracentrifugation at 100,000 × g for 120 min at 4°C to separate membrane fraction (pellet) and soluble fraction (supernatant). The cell wall and membrane fractions were washed once and then re-suspended in PBS. Total protein content of all fractions was measured and 20 μg of protein was processed for SDS-PAGE (12%) Inner membrane, outer membrane, periplasmic, and cytosolic fractions of TlyA expressing *E. coli* were prepared as reported earlier using sucrose density gradient approach (Wai et al., 2003b).

### Immunoblotting

Whole-cell lysates of bacterial cultures were prepared as described previously and analyzed by SDS-PAGE (12%) for immunoblotting. Mouse anti-serum against TlyA was used at a dilution of 1:10000, and monoclonal antibodies of anti-GroEL (BEI resources, NR-13813) and anti-HBHA (BEI resources, NR-13804) at 1:500 dilutions. The anti-mouse peroxidase-conjugated antibody (#7076 cell signaling) was used as a secondary antibody. The *E. coli* specific anti-GroEL (Abcam ab-82592) and anti-β-lactamase (Abcam ab-12251) antibodies were used at a dilution of 1:500.

### Isolation of membrane vesicles (MV) and outer membrane vesicles (OMV)

MV from *M. smegmatis* and OMV from *E. coli* cultures were isolated as described previously (Wai et al., 2003a; Prados-Rosales et al., 2011). TlyA transformed bacteria, were harvested at OD_600_~0.8 by centrifugation at 7000 × g for 10 min at 4°C. The supernatant was filtered through a 0.45 μm-pore size membrane filter. An aliquot of the supernatant was re-plated on an appropriate 7H10/LB-antibiotic plate to ensure the absence of viable bacteria. The cell free supernatant was centrifuged at 150,000 × g for 2 h at 4°C using the TLA-100 rotor. The vesicle pellets were washed once and then re-suspended in PBS and stored in −20°C for all experiments.

### Immuno gold labeling of intact bacteria

Negative staining of bacteria for electron microscopy was carried out as described earlier (Wai et al., 2003a; Elluri et al., 2014). Briefly, non-transformed and TlyA transformed *M. smegmatis* were washed several times with PBS, and re-suspended in the same buffer followed by incubation with immune rabbit serum raised against TlyA (1:100) for 1–2 h on ice. After washing thrice with PBS, the samples were further incubated with anti-rabbit IgG-gold (10 nm, Sigma-Aldrich G-7402) for 1 hr. The unbound gold conjugated antibody was removed by extensive washing with PBS. The samples were negatively stained with 2% Uranylacetate on carbon coated grids for visualization in Jeol electron microscope.

### Immunogold labeling MV and OMV

MV and OMV samples were isolated as described above. The specimens for electron microscopy were prepared by incubation of an aliquot of OMV preparation (100 μl) with TlyA specific immune rabbit serum (1:100 dilution) in PBS for 1 h on ice. The vesicles were separated from the serum by centrifugation at 150,000 g for 2 h at 4°C, and washed three times with PBS. The resultant vesicle fraction was mixed with a colloidal gold conjugated antibody (Sigma-Aldrich G-7402) and kept on ice for 1 h and unbound gold particles were removed by washing. The final vesicle preparation was negatively stained with 2% Uranylacetate on carbon coated grids and examined in Jeol electron microscope (Elluri et al., 2014).

### Statistical analysis

The results are presented as the mean ± S.D. Statistical significance was calculated using Student *t*-test.

## Results

### Endogenous TlyA of *M. smegmatis* is not detectable

The data presented in Supplementary Figure 1A shows the effect of inducer, Acetamide, on the expression of MtbTlyA in *M. smegmatis*. In order to keep low expression threshold, we have set the inducer concentration at 0.001% for all the experiments described here. This concentration of inducer is about 200 fold lower in comparison to the normal protein expression in *M. smegmatis* (Noens et al., 2011). The MtbTlyA can be purified to homogeneity (Supplementary Figure 1B) and its activity exhibits concentration dependence (Supplementary Figure 1C). The activity assays were carried out as described earlier (Rahman et al., 2010).

*M. smegmatis* was annotated to contain a *tlyA* gene product, referred here as MsTlyA (MSMEG_3751) represented by, A0QYR0 (UniProtKB) which has ~75% identity with the MtbTlyA. However, its existence status (as on Mar 2015) is “predicted” and no experimental evidence exists for its expression. A deeper examination of ortho-proteogenomic approaches (covering over 900 proteins), cell-wall specific proteomic studies (covering over 300 proteins) as well as of proteomic response to various drugs by *M. smegmatis* (covering over 2500 proteins) could not detect any peptide corresponding to the MsTlyA using a variety of mass-spectrometry approaches (Wang and Marcotte, 2008; Gallien et al., 2009; He and De Buck, 2010). We therefore, sought to examine the status of endogenous MsTlyA by RT-PCR for *tlyA* transcript in *M. smegmatis*, immuno blot-detection of whole cell lysate and LC-MS analysis of whole cell lysate for the MW range ~21–45 KD (boxed region of Figure 1A). We could not detect any band corresponding to MsTlyA (Figure 1B) or in membrane fraction (Figure 1C). We have also not detected the *tlyA* transcript despite repeated attempts under varying conditions (Figure 1D). This is consistent with an earlier observation in which the *tlyA* gene was said to be absent in *M. smegmatis, M. vaccae, M. kansasii, M. chelonae*, and *M. phlei* based on PCR amplification. Moreover, southern hybridization using the same PCR product of *M. tuberculosis* could not identify any positive band in *M. smegmatis* (Wren et al., 1998). In support of these observations, our proteomic attempt also could not detect any peptide(s) corresponding to MsTlyA either in whole cell lysate or enriched membrane fractions (The list of protein identified by us are detailed in Supplementary Tables 1–3). We could easily detect several peptides of MtbTlyA upon expression in *M. smegmatis* (Supplementary Table 2). This proteomic attempt of *M. smegmatis* has allowed us to confirm the expression of 40 proteins in varying conditions and the identity of these proteins is listed in Supplementary Table 4, which may help other investigators. In essence, we could not positively ascertain the expression of endogenous TlyA of *M. smegmatis*. It is relevant to mention here that Akhilesh Pandey and co-workers have detected three peptides of MtbTlyA, notably, in the culture filtrate of H37Rv (Kelkar et al., 2011).

### TlyA is present on the cell-surface

While evidences in the literature indirectly suggested the presence of “hemolysin like” molecules at the cell wall of *M. tuberculosis*, there had been no direct visualization of TlyA to date. In view of this, we next examined for the cell surface presence of TlyA by immuno-fluorescence on the surface of wild type *M. marinum*, TlyA expressing *M. smegmatis* and *E. coli*. Our confocal visualization showed clear staining for the TlyA on wild type *M. marinum* (Figure 1E), *M. smegmatis* (Figure 1F) and also on the surface of *E. coli* (Figure 1G). We have also performed dual staining of TlyA expressing *M. smegmatis* with anti-HBHA protein which is known to be part of cell-wall. As shown in Figure 1H (top panel), the TlyA is colocalized with HBHA. It is relevant to mention here that our staining procedures did not involve either permeabilization or fixation. For example, the GroEL of *M. smegmatis* cannot be visualized without permeabilization (Figure 1H, middle panel) while it can be seen only after permeabilization (Figure 1H bottom panel). These results suggest us that our staining procedures are not detecting the TlyA that is intracellular.

### TlyA is a surface-accessible protein

**Figure 2:**
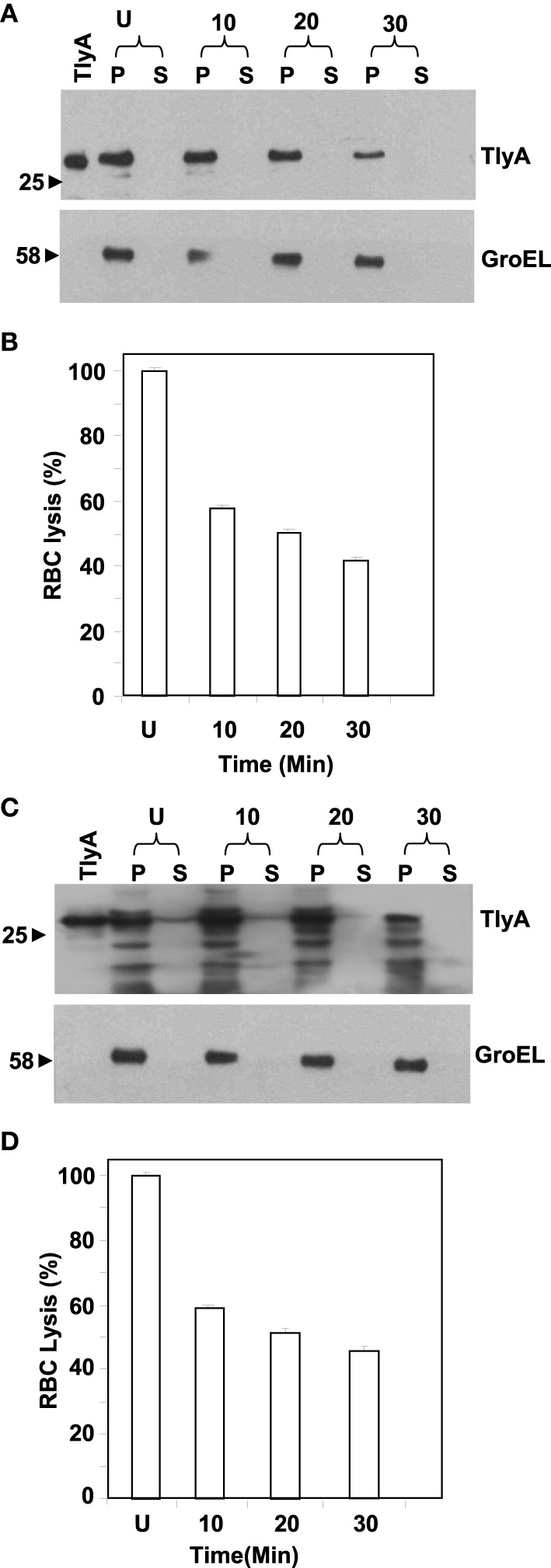
**Susceptibility to extrinsic proteases of TlyA expressing ***M. smegmatis*** (A,B) and ***E. coli*** (C,D):** *M. smegmatis*/TlyA **(C)** and *E. coli*/TlyA **(D)** were treated with Proteinase K and the resultant samples were processed for 12% SDS–PAGE and detected with anti-TlyA antibody. P and S indicate pellet and supernatant obtained at indicated times in minutes. The antibodies used for the detection of the blot are indicated on the right side. The lane marked with TlyA indicates purified TlyA as control. The **(B,D)** respectively represent the hemolytic activity of the Proteinase K treated bacteria, shown in **(A**,**C**). The labels are indicated below the bars. Proteinase K treated bacteria.(7 × 10^7^) were incubated with 1% rabbit RBC at room temp for 24 h to assess the degree of lysis by measuring the absorbance at 540 nm of RBC free supernatant.

We next examined for exposed amino acid segments of TlyA upon expression in *M. smegmatis* and *E. coli* with the help of limited proteolysis by Proteinase K. In this approach, we have incubated the bacteria expressing the TlyA with and without Proteinase K and equal aliquots of bacteria were then analyzed by SDS-PAGE followed by immunoblotting. In principle, even small surface-exposed loops/amino-acid segments are expected to be cleaved upon exposure to Proteinase K. As shown in Figures 2A,C, the intensities of the bands of the full-length TlyA, associated with *M. smegmatis* (Figure 2A) and *E. coli* (Figure 2C), were reduced by more than 50% upon Proteinase K treatment. In contrast, the intensity of the intracellular GroEL remained constant over the same period implying that the Proteinase K treatment of the bacteria has not resulted in digestion/degradation of intracellular proteins. Consistent with this observation, the Proteinase K digestion has diminished the contact dependent hemolytic activity of the *M. smegmatis* (Figure 2B) and *E. coli* (Figure 2D) associated TlyA, which is a characteristic property of TlyA expressing bacteria (King et al., 1993; Wren et al., 1998; Rahman et al., 2010). It is clear from the figure that the hemolysis has significantly reduced in 10 min. Thus, it is likely that the amino-acid segments of TlyA are exposed at the bacterial surface of *M. smegmatis* and *E. coli* and accessible to Proteinase K.

### Cell surface presence of TlyA is not dependent on SecA2, TatA and TatC

Both Tat and Sec pathway transport proteins which have characteristic signal sequence at their N- terminal for secretion (Lee et al., 2006). The SecA2 is responsible for the export of a small subset of proteins and is required for virulence of the pathogenic *M. tuberculosis* (Braunstein et al., 2003; Gibbons et al., 2007). *M. smegmatis* also possesses a SecA2 system, which is functionally conserved with that of *M. tuberculosis* (Braunstein et al., 2001, 2003). In *M. tuberculosis* it has not been possible to construct Tat deficient mutants which appears to be essential (Saint-Joanis et al., 2006; McDonough et al., 2008). It is relevant to note that the TlyA does not have any signal sequence in favor of either Sec or Tat pathways. If the TlyA was to utilize either of these two pathways through non-conventional or pseudo motifs, the Tat/Sec pathway knock-out strains of *M. smegmatis* should not show the presence of TlyA in the membrane fractions. The TatA, TatC, SecA2 deficient strains of *M. smegmatis* used in the present are same as the ones reported earlier (McDonough et al., 2005, 2008; Gibbons et al., 2007). We, therefore, have carried out the fractionation of the Δ*TatA*, Δ*TatC*, Δ*SecA2* expressing the TlyA construct by using well established procedures (Gibbons et al., 2007).

**Figure 3:**
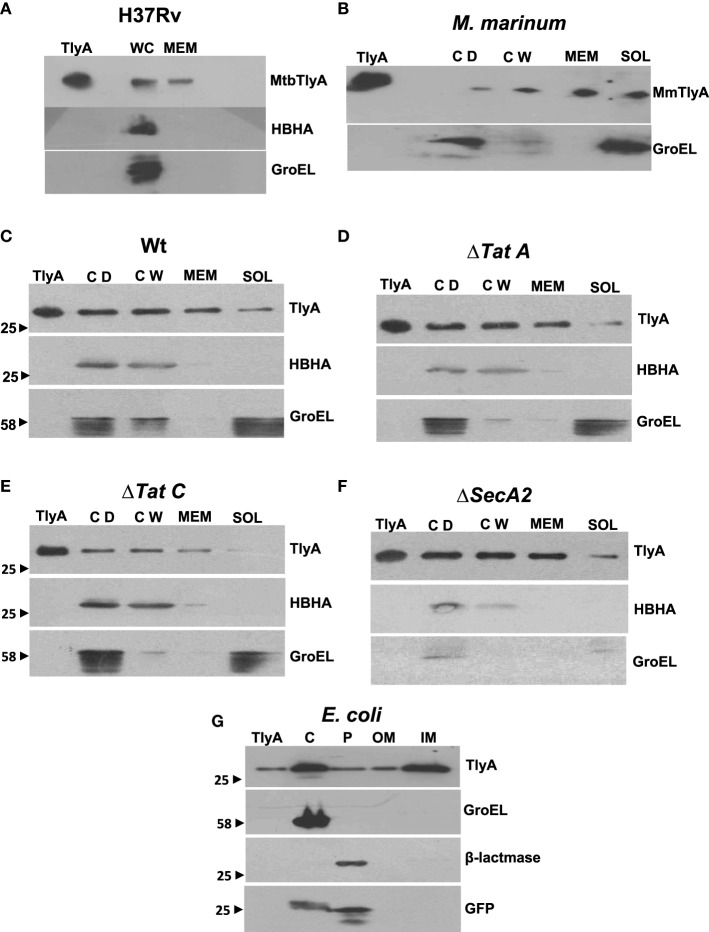
**Subcellular fractionation of ***M. tuberculosis*** (A) ***M. marinum*** (B), TlyA expressing ***M. smegmatis*** WT (C), Δ***TatA*** (D), Δ***TatC*** (E), Δ***SecA2*** (F), and ***E. coli***/TlyA (G):** The bacterial strains are indicated at the top of each panel. For all panels, the antibodies used for detection are indicated on the right side. Purified TlyA from *E. coli* was used as a marker for identification of TlyA band in all blots. The samples used for **(A)** were obtained from BEI resources. WC and MEM represent whole cell lysate and membrane fraction respectively. In **(B–F)**, the lane markings CD, CW, MEM, and SOL respectively represent cell debris obtained after 3000 g, cell-wall after 27,000 g and membrane pellet fraction obtained after 100,000 g and soluble fraction of 100,000 g (supernatant). In panel G, the C, P, OM, and IM respectively represent the cytosolic, periplasmic, outer-membrane, and inner-membrane fractions. The panels are a representative of one of the three independent experiments.

The data in Figures 3A,B respectively show the presence of MtbTlyA and MmTlyA in the membrane fractions of *H37Rv* and *M. marinum* respectively. It is relevant to mention here that the whole cell lysate and membrane fractions, shown in Figure 3A, were obtained from BEI resources and serve as an independent verification. As expected, the MmTlyA is also detected in the membrane fraction as seen Figure 3B.

**Figure 4:**
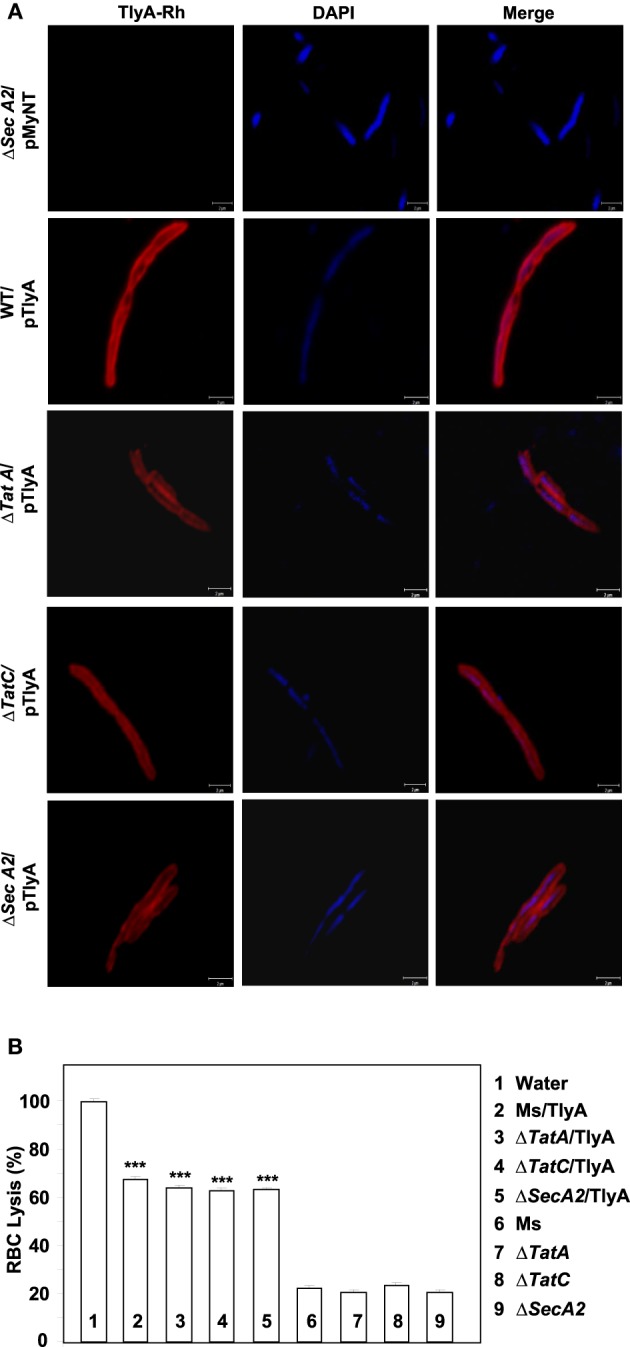
**(A) Immunofluorescence visualization of TlyA in Δ***TatA***, Δ***TatC*** and Δ***SecA2*** deficient strains of ***M. smegmatis***:** TlyA expressing Δ*TatA*, Δ*TatC* and Δ*SecA2* deficient strains were immuno-stained with anti-TlyA antibody and visualized with Rhodamine conjugated anti-rabbit antibody IgG. Left side panels show the staining for TlyA (red). Middle panels show the DAPI staining of bacteria (blue) and the right panels show the merged with red and blue channels. The bar represents 2 μm. The panels are a representative of one of the three independent visualizations. **(B) Contact dependent hemolysis of TlyA expressing Δ***TatA***, Δ***TatC*** and Δ***SecA2*** deficient strains of ***M. smegmatis***:** TlyA transformed and non-transformed *M. smegmatis* (2 × 10^7^) were incubated with 1% rabbit RBC at room temp for 24 h to assess the degree of lysis, in comparison to the water lysed RBC (bar marked with 1) by measuring the absorbance at 540 nm of RBC free supernatant.

The immunoblot in Figure 3C shows an unambiguous presence of MtbTlyA in the membrane fraction of *M. smegmatis* upon its expression. Interestingly, the MtbTlyA is also seen in the membrane fractions of *M. smegmatis* deficient in Δ*TatA* (Figure 3D), Δ*TatC* (Figure 3E) and Δ*SecA2* (Figure 3F). It is relevant to note that SecA1 deficiency is lethal for *M. smegmatis* and hence, could not be studied (Braunstein et al., 2001). We have also examined for HBHA and GroEL as internal controls of this fractionation. While the HBHA is always restricted to cell-wall fraction, the GroEL is found only in soluble fraction than membrane fraction, which validates our fractionation attempt. After having visualized the TlyA in *M. smegmatis*, we next performed sub-cellular fractionation of *E. coli*/TlyA to separate outer-membrane, periplasmic, inner-membrane, and cytosolic fractions by well established protocols (Wai et al., 2003b). The TlyA is present in inner-membrane, periplasmic space and outer membrane (Figure 3G). Under the same conditions, the GroEL, β-lactamase and GFP were observed in cytosolic, periplasmic and cytosolic fractions which again support our fractionation attempt. The small periplasmic presence of GFP could be due to osmotic shock in the procedure. Hence, the presence of TlyA in the membrane fractions of *M. smegmatis* or *E. coli* is not due to artifact of expression but a specific sequestration might be responsible since membrane fractions of both H37Rv, *M. marinum* also exhibit an unambiguous presence of TlyA. In support of all the above observations, we could easily see the presence of TlyA on the surface of Δ*TatA*, Δ*TatC*, and Δ*SecA2* deficient strains of *M. smegmatis* while the mock vector-transformed bacteria did not exhibit any positive fluorescence staining as seen in Figure 4A In support of this observation, all the TlyA expressing Δ*TatA*, Δ*TatC*, and Δ*SecA2* knockout strains have exhibited contact dependent hemolytic activity (Figure 4B). The data shown in Figure 4B clearly let us infer that there is no significant difference in hemolytic activity among the TlyA expressing strains. Hence, the TlyA does not seem to depend on either Tat or Sec pathways for translocation to cell-wall *of M. smegmatis*.

### TlyA are part of membrane and outer membrane vehicles (MV and OMV)

**Figure 5:**
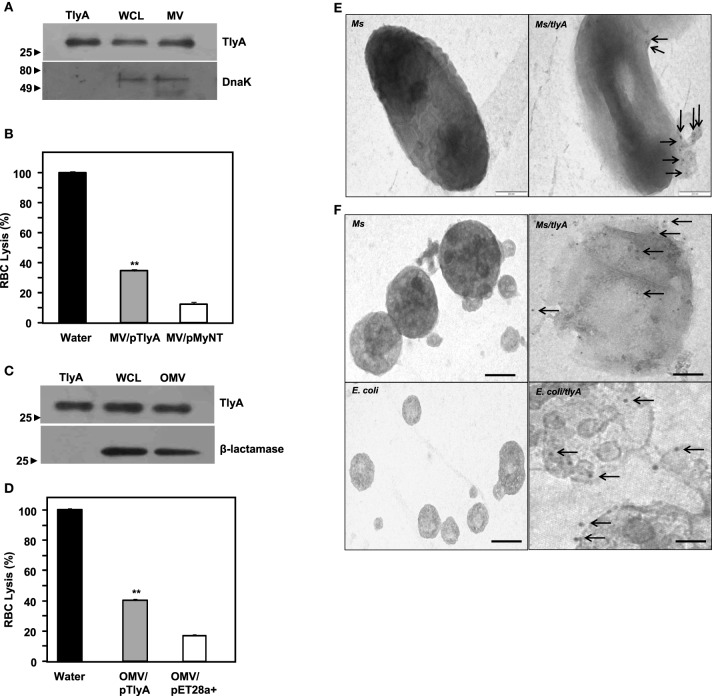
**Presence of TlyA in Membrane Vesicles (MV) of ***M. smegmatis*** (A,B) and Outer Membrane Vesicles (OMV) of ***E. coli*** (C,D):** The MV secreted by *M. smegmatis* or OMV secreted by *E. coli* expressing the TlyA were obtained as described in methods section. The presence of TlyA in the vesicles was ascertained by developing respective blots with anti-TlyA antibody **(A,C)**. Hemolytic activity of MV secreted by *M. smegmatis* **(B)** and OMV secreted by *E. coli* **(D)** was obtained by mixing 20 μg/ml (total protein) of vesicle preparation with 1.5% rabbit RBC. After 24 h of incubation, the absorbance was measured at 540 nm for release for hemoglobin. **EM visualization of OMVs: (E)** Electron micrograph of a plain and TlyA transformed *M. smegmatis* (top right panel) and non-transformed *M. smegmatis* is shown in top left panels that wee stained with 10 nm gold labeled antibody. The vesicle attached to the intact bacterium is shown marked with arrow. The **(F)** represents the electron micrograph of a purified MV and OMVs. Both MV and OMV show significant deposition of gold particles as shown in the right panels (marked with arrows) while the left panels show no gold particles which represent the MV and OMV of non-transformed bacteria. In all panels the magnification bar represents 200 nm.

Both gram negative and gram positive bacteria secrete Outer Membrane Vesicles (OMV) and Membrane Vesicles (MV) respectively (Wai et al., 2003a; Prados-Rosales et al., 2011). We, therefore, examined whether we can detect TlyA in MV secreted by *M. smegmatis* and OMV secreted by *E. coli*. The supernatants used for the identification of TlyA in the membrane vesicles was thoroughly assessed for the absence of viable bacteria by plating an aliquot on appropriate plate after filtration through 0.45 μm membrane. As shown in Figure 5A (MV from *M. smegmatis*) and Figure 5C (OMV from *E. coli*), the presence of TlyA is unambiguous in both MV and OMV fractions. In this regard, we have used DnaK in case of MV from *M. smegmatis* and β-lactamase in case of OMV from *E. coli* to authenticate our vesicle preparations. Both MV and OMV shown above contain active TlyA since they are able to lyse rabbit RBC as seen in Figures 5B,D. We have also attempted to examine these vesicles by negative staining in transmission electron microscopy. We have found small outer membrane vesicles surrounding the intact bacterium with gold particles (Figure 5E right panel) while the non-transformed bacteria did not exhibit any gold particles on its periphery (Figure 5E left panel). We have also examined the purified MV and OMV by immune-gold labeling and negative staining. The EM micrographs of MV of *M. smegmatis*/TlyA (Figure 5F top right panel) and OMV of *E. coli*/TlyA (Figure 5F bottom right panel) have shown gold particles at the periphery of the vesicles while the non-transformed MV (Figure 5F top left panel) or OMV (Figure 5F bottom left panel) did not exhibit any gold particles in their periphery. These observations suggest that the bacterially expressed TlyA is capable of reaching the extra-cellular milieu using a vesicle mediated transport.

## Discussion

The goal of this study is to ascertain whether or not the TlyA class of proteins can reach extra-cellular milieu despite the absence of signal sequences. The TlyA, currently annotated as a rRNA methyl transferase, can also function as a hemolytic entity. In order to exert its hemolytic functionality, it is necessary for the TlyA to establish a physical contact with the target membrane for this “non-conventional hemolytic trait.”

We have presented several evidences which show the presence of TlyA on the surface of *M. marinum* (native host), *M. smegmatis* (a surrogate host) and *E. coli* (a heterologous host) (Figure 1). The presence of TlyA at the cell-wall of these bacteria was strengthened with data on protease accessibility as shown in Figure 2. The TlyA protein was also detected in the membrane fraction of H37Rv (Figure 3A), *M. marinum* (Figure 3B), *M. smegmatis* (Figure 3C) and *E. coli* (Figure 3G). It is relevant to note that Kelkar et al. have also detected 3 peptides of TlyA in the culture filtrate by mass spectroscopy (Kelkar et al., 2011). Interestingly, the TlyA is not dependent on TatA, TatC or SecA2 pathways to reach the cell-surface. Although it might appear that the proportion of TlyA present in TatC knockout strain is relatively less in comparison to other strains but it is not completely absent and it can be visualized on the surface of the bacteria (Figure 4A). These observations suggest us that the TlyA may not utilize the Tat or Sec mediated transport mechanism to reach the cell-surface. At the outset it might appear that the TlyA may utilize the Esx-1 secretion pathway of mycobacterial species. However, it is known that the Esx-1 secretion pathway of *M. smegmatis* is involved in conjugation rather than protein secretion (Flint et al., 2004). Moreover, proteins secreted through the mycobacterial Esx-1 system appear to contain a conserved sequence at their C-terminus *viz*. “WXG” with a size limit of < 100 amino acids. The TlyA is not fulfilling either of the criteria since it has 268 amino acids and does not contain any “WXG” motif in its C-terminal region. In addition, the TlyA does not seem to have any “partner” to facilitate the secretion through dimerization as the well known Esx-1 substrates *viz*. Esat6 and Cfp10 are secreted as hetero-dimer. In view of these observations, we speculate that the TlyA may not be utilizing the Esx-1 pathway. It is relevant to mention here that the mycobacterium possesses many proteins which do not have the classical signal sequences that signify the Tat or Sec pathways (listed in Table 2), yet, found to be present at the cell surface. Hence, more studies are needed to identify the routes they utilize to reach the cell-surface and their function. In essence, the TlyA can reach the bacterial cell-wall despite the absence of signal sequence or apparent transmembrane segments identifiable with the present knowledge embedded in Bioinformatic tools.

**Table 2:** **List of ***M. tuberculosis*** proteins present on its cell wall without any classical signal sequence**.

**Protein**	**Gene symbol**	**Software**			**Gravy**	**Technique**	**References**
		**Tat**		**Sec**			
		**Tat P**	**Tat Find**	**Signal P**			
Rv2560	*pknJ*	–	–	–	0.488	^a^IEM	Plaza et al., 2007
Rc2707	–	–	–	–	0.516	IEM	Cowley et al., 2004
Rv1490	–	–	–	–	0.759	IEM	Patarroyo et al., 2008a
Rv2004c	–	–	–	–	0.157	^b^CF/^c^TEM	Forero et al., 2005
Rv2969c	–	–	–	–	0.106	CF/IEM	Patarroyo et al., 2008b
Rv1694	*tlyA*	–	–	–	0.088	^d^IF/CF	Kelkar et al., 2011

a *IEM, Immuno electron microscopy*.

b *CF, Culture Filtrate*.

c *TEM, Transmission Electron microscopy*.

d *IF, Immuno fluorescence, this study*.

The hemolytic trait of the MtbTlyA discussed here is also in agreement with certain instances reported in the literature. For example, the TlyA of *H. pylori* (our unpublished data and recently published work of Lata et al., 2014) and a recent study involving TlyA of *L. interrogans* have all showed hemolytic activity, while TlyA of *L. interrogans* needs a formal confirmation for its rRNA methylase activity (Rahman et al., 2010; Wang et al., 2012). Although, we have earlier ruled out the role of other molecules for the hemolytic activity, it is still possible that other proteins and/or complexes might be involved for the said hemolytic activity. It is also possible that not all TlyA proteins (identified so far in literature in various species) may exhibit this hemolytic trait as it may also depend on the environment. For example, the TlyA of *H. pylori* was found to be restricted to whole bacterial cells and insoluble fractions (membrane fractions) based on hemolytic activity suggesting that the TlyA is somehow associated with cell-wall (Martino et al., 2001). Interestingly, the TlyA is also detected in the membrane vesicles secreted by *M. smegmatis* and *E. coli* (Figure 5). In comparison to TlyA protein, the *E. coli* ClyA (UniProtKB:P77335) also sequesters into inner membrane, outer membrane vesicles despite the absence of Tat or Sec signal sequences (Wai et al., 2003a,b). Though TlyA and ClyA are not homologous, both appear to get exported through outer membrane vesicles in a similar fashion.

We have recently shown that TlyA expressing bacteria adhere better to RAW264.7 macrophages and get phagocytosed efficiently. The internalized bacteria avoid acidification to the extent of >65% in case of *E. coli* and >80% in case of *M. smegmatis* (Mittal et al., 2014). Moreover, both spleen and lung of TlyA immunized mice have exhibited four fold less CFU in comparison to the unimmunized mice when the mice were challenged with TlyA expressing *M. smegmatis*, while the CFU of immunized mice challenged with normal *M. smegmatis* have remained same as that of the unimmunized mice. This result suggests that the TlyA is exposed on the surface of the *M. smegmatis* and immune serum can recognize it under *in vivo* conditions (Mittal et al., 2014). In summary, TlyA can reach the cell-wall of bacteria that express it despite the absence of signal sequences. It is interesting to study how the cell-wall sequestration is orchestrated by the bacteria and the factors that contribute to it.

### Conflict of interest statement

The authors declare that the research was conducted in the absence of any commercial or financial relationships that could be construed as a potential conflict of interest.
