# Supplementary figures and images for: Mycobacterial tlyA gene product is localized to the cell-wall without signal sequence
Source: Front Cell Infect Microbiol. 2015 Aug 21;5:60. doi: 10.3389/fcimb.2015.00060 (PMC4543871; doi:10.3389/fcimb.2015.00060)

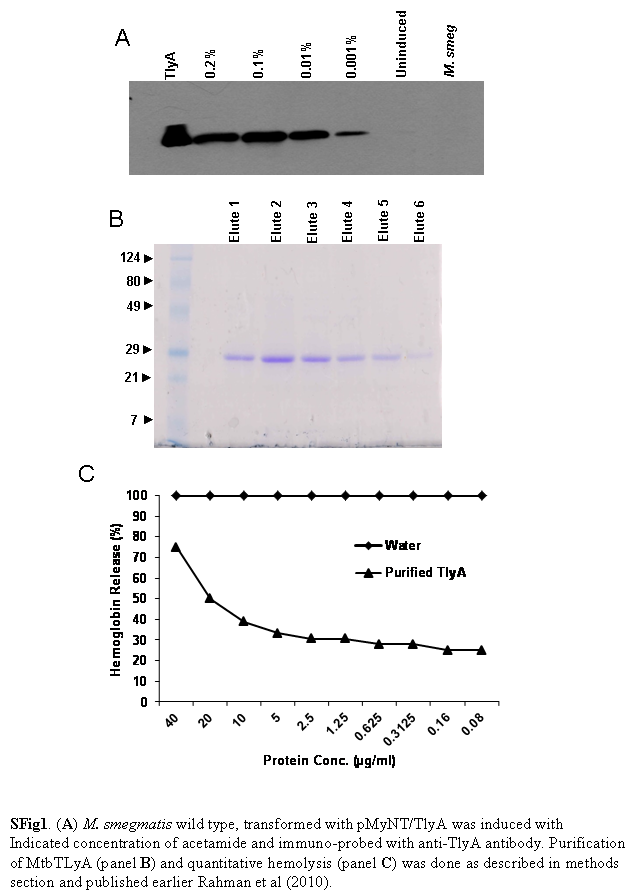

Supplement: Supplementary file 5 [file Image1.TIF]
